# Interaction of occupational manganese exposure and alcohol drinking aggravates the increase of liver enzyme concentrations from a cross-sectional study in China

**DOI:** 10.1186/1476-069X-12-30

**Published:** 2013-04-15

**Authors:** Qi Deng, Jing Liu, Qing Li, Kangcheng Chen, Zhenfang Liu, Yuefei Shen, Piye Niu, Yiping Yang, Yunfeng Zou, Xiaobo Yang

**Affiliations:** 1 Department of occupational health and environmental health, School of Public Health, Guangxi Medical University, Guangxi, Nanning, China; 2 School of Public Health, Guangxi Medical University, Guangxi, Nanning, China; 3 Department of Hematology, The First Affiliated Hospital of Guangxi Medical University, Guangxi, Nanning, China; 4 Department of Neurology, The First Affiliated Hospital of Guangxi Medical University, Guangxi, Nanning, China; 5 Department of Occupational health and environmental health, School of Public Health and Family Medicine, Capital Medical University, Beijing, China; 6 Center for Genomic and Personalized Medicine, Guangxi Medical University,, Guangxi, Nanning, China

**Keywords:** Manganese exposure, Drinking, Liver enzymes, Interaction

## Abstract

**Background:**

Over exposure to manganese (Mn) can damage the human central nervous system and potentially cause liver toxicity. Alcohol drinking is also one of the well-known harmful factors to hepatic organism. The interaction between Mn exposure and alcohol consumption to liver function was investigated in this study.

**Methods:**

A total of 1112 on-the-spot workers were included in the cross-sectional survey from a large scale of manganese exposed workers healthy cohort (MEWHC) in a ferro-manganese refinery company. A questionnaire was used to collect the demographic information, occupational history, and alcohol drinking habits. Occupational health examination was carried out for each worker. The five key serum indices, including total bilirubin (TBILI), direct bilirubin (DBILI), indirect bilirubin (IBILI), alanine transaminase (ALT), and aspartate transaminase (AST), were determined to evaluate the liver function of each subject.

**Results:**

Workers exposed to high levels of Mn had significantly elevated serum concentrations of liver enzymes (DBILI: 3.84±1.20 μmol/L, ALT: 27.04±19.12 IU/L, and AST: 29.96±16.68 IU/L), when compared to those in the low-exposure group (DBIL: 3.54±0.85 μmol/L, ALT: 20.38±10.97 IU/L, and AST: 26.39±8.07 IU/L), all *P*<0.01. These serum indices had a significantly increasing trend with the elevation of Mn exposure level (*P*_trend_ <0.01). In addition, the workers with alcohol drinking also showed higher concentrations of liver enzymes than those non-drinkers, especially, and there was significant interaction between Mn exposure and alcohol consumption in terms of these three indices (*P*<0.001).

**Conclusions:**

Occupational exposure to Mn can lead to a dose-dependent increase of liver enzyme concentrations, and interact with alcohol drinking to potentially aggravate the liver damage. It will be important for Mn exposed workers to control drinking and also assess liver function in the occupational health examination.

## Introduction

Manganese (Mn) is an essential trace element for humans, but long-term occupational exposure to Mn including the manufacturing of batteries and ferroalloy, smelting, welding, and mining, mainly leaded to health damage of central nervous system and lungs [1,2]. Serious manganism cases which were rarely reported recently, mainly displayed neurological system dysfunction like Parkinson's disease (PD) [3-5], but its toxicity to other organs may deserve more attention for researchers.

Liver is the most important one of the major organs involved in the storage, biotransformation and detoxification of toxic substances, was of interest in heavy metal poisoning [6]. Manganism and hepatic encephalopathy were the most common pathologies associated with the effects of Mn exposure [7]. Metal Mn had a special affinity to mitochondrial and was mainly stored in those tissues with rich mitochondrion. For example, the accumulation of Mn in the mitochondria of brain was associated with neurological symptoms of manganism *in vivo*[8,9]. Liver is also rich of mitochondria, which is obviously the target organ of Mn accumulation. Earlier reports explained the pathogenetic mechanism of manganism, which mainly had the influence exerted directly on the internal liver organs [10]. Animal experiments also showed that mice exposed to Mn displayed the Mn accumulation and damage in the liver and kidney [11].

Alcohol is also one of well-known harmful factors to liver. Workers engaged in high working stress situation are usually more likely to have higher alcohol drinking habits or frequency. In order to explore the toxicity of Mn and its interaction with alcohol consumption on the liver function, a cross-sectional health survey was performed based on our large scale of Mn smelting exposure workers healthy survey cohort (MEWHC) in China.

## Methods

### Study design and subjects

The cross-sectional epidemiological study was one part of baseline survey of our prospective MEWHC design from July to October 2011. This cohort would be composed of nearly 2000 workers with chronic occupational Mn exposure in the future, and the total baseline survey of this cohort study was progressively finished at the end of 2012. The main purpose of this cohort was to explore the early-stage or long-term healthy effects of Mn exposure on different organs and tissues, and especially explore the genetic susceptibility among individuals. During the first stage of this cohort, we investigated a total of 1112 workers who were chronically exposed to Mn and included in the present survey. All subjects were recruited from one large ferro-Mn alloy production plant in Guangxi, China. The inclusion criteria for participation were: age more than 18 years, working time no less than 3 months, and no physical disability or surgical history. The exclusion criteria included history of medication and drug use in the past two weeks, diagnosis as chronic hepatitis B patients, and no willingness to participate the interviews. This study was approved by the Ethics and Human Subjects Committee of the Guangxi Medical University.

### Questionnaires and occupational health examination

A structured questionnaire was used to collect data on demographic information, occupational history, medication history and especially drinking habit. Smoking habit was defined as smoking (smoking at least one cigarette daily for more than three months), no smoking (never or former smoking with cessation for more than three months). Drinking habit was defined as current drinking (drinking at least once each week for more than three months) and no drinking (never or former drinking). The recent medication history included taking a medication in the past two weeks and taking drugs that had potential influences on liver function. After obtaining written informed consent, trained interviewers used this questionnaire to collect epidemiological data during face-to-face interview following the structured guidelines.

The standardized exam was strictly performed based on those items of general chart of occupational health examination including symptoms, clinical signs, and some special tests like lung function, which were generally required by Chinese law and regulations. All doctors must be qualified to carry out occupational health examination, especially, and the examining physician always served these occupational workers in the past years and had sufficient expertise to diagnose manganism or even signs of Parkinsonism. The occupational health examination was performed in the morning at the end of the work shift after overnight fasting. Data of abnormal clinical signs were collected in independent rooms by qualified physician and surgeon, respectively. Finally, each participant donated 5.0 ml venous bloods used to laboratory testing.

### Monitoring of air Mn concentration

Environmental exposure information was obtained through workplace monitoring in this Mn smelter. The factory provided its daily monitoring data of Mn concentration in various workplaces in the past years. The monitoring targets included three smelting branch factories in this ferro-Mn alloy plant, and a total of six workshops. Air sampling method was conducted according to the standard specification issued by Ministry of Health in China "Specifications of Air Sampling for Hazardous Substances Monitoring on the Workplace"(GBZ159-2004). To avoid the impact of confounding factors, the sampling must be carried out in normal working conditions. Those air samples were firstly digested with 5 mL of HClO_4_–HNO_3_ mixture (1:9 v/v) at temperature 200°C, and detected in 279.5 nm wavelength using by a model HITACHI Z-5000 acetylene-air flame atomic absorption spectrophotometer (AAS).

### Determination of blood chemistry indices

A total of five key serum indices, including serum total bilirubin (TBILI), direct bilirubin (DBILI), indirect bilirubin (IBILI), alanine transaminase (ALT), and aspartate transaminase (AST), were detected to represent the hepatic function status together. TBILI, DBILI, and IBILI were measured using Jendrassik and Grof’s (J-S) method, and the Rate method was used to determinate serum AST and AST concentrations. All of the described methods were adapted for automated analysis by Hitachi 7020 automatic biochemical analyzer from Japan.

### Statistical analyses

All statistical analyses were performed using the SPSS version 17.0 program. Normal distribution was evaluated by determination of skewness and plotting of values as histograms. The general demographic characteristics and anomaly clinical signs in our MEWHC population were showed with absolute number and proportion in each subgroup. Analysis of covariance (ANOVA) was used to compare the differences of these indices between different exposure subgroups with adjustment gender, working years and drinking habits. Pearson correlation analysis was used to calculate the varying trend of liver function indices with exposure dose, and the general linear model were used to identify those factors associated with the liver function damage and also interaction between drinking and Mn exposure. All statistical tests were two-tailed and statistical significance was defined as *P* < 0.05.

## Results

### Demographic characteristics

As shown in Table 1, the present investigated populations from one part of MSWHC totally contained 1112 workers. There were 768 male workers (69.1%) and 344 female workers (30.9%). The average age were 39 years old, and the average length of work was 13 years. Especially, 773 workers (69.5%) were current drinkers, and 563 workers (50.6%) were current smokers. None of the study subjects had ever been diagnosed as occupational diseases according to the diagnostic criteria in China.

**Table 1:** Demographic characteristics of the manganese exposed workers healthy cohort (MEWHC)

**Variable**	**Number**	**Percent (%)**
Total	1112	100.0
Sex		
male	768	69.1
female	344	30.9
Length of service(years)		
≤10	491	44.2
10~20	340	30.6
≥20	281	25.3
Grouping by exposure levels		
Low-exposure^a^	381	49.5
Intermediate-exposure^b^	181	16.3
High-exposure^c^	550	34.3
Alcohol drinking status		
Drinker	773	69.5
Non-drinker	339	30.5
Smoking status		
Smoker	563	50.6
Non-smoker	549	49.4
Marry status		
Married	935	84.1
Never married	177	15.9
Education status		
Junior school or below	579	52.1
High school or above	533	47.9

^a^ Low-exposure group includes those workers in the non-production workplaces such as drivers or cookers, and air Mn concentration ranged in 0~0.02 mg/m^3^.

^b^ Intermediate-exposure group includes those workers in the auxiliary workshops such as weld workers or ingredient workers. The air Mn concentration ranged in 0.07~0.15 mg/m^3^ before 2007, and in 0.02~0.14 mg/m^3^ after 2007.

^c^ High-exposure group only includes smelting workers. The air Mn concentration ranged in 0.36~1.06 mg/m^3^ before 2007, and in 0.08~0.45 mg/m^3^ after 2007.

All workers were divided into three exposed groups according to the working position in this factory. Low-exposure group included those workers in the non-production workplaces, such as drivers or cookers who had little exposure opportunity. Intermediate-exposure group included those in the auxiliary workshops such as welding workers or ingredient workers. High-exposure group only included those smelting workers who were exposed to high air concentration of Mn in the production workshops.

### Air concentrations of Mn

From 2006 to 2008, some technical innovations were gradually carried out to improve the working air environment in this ferro-Mn alloy factory. The monitoring results showed that the air concentrations of Mn significantly decreased after 2007. The concentrations in the auxiliary workshops ranged in 0.07~0.15 mg/m^3^ before 2007, and in 0.02~0.14 mg/m^3^ after 2007. In smelting production workshops, the air concentration of Mn ranged in 0.36~1.06 mg/m^3^ before 2007, and then decreased in 0.08~0.45 mg/m^3^ after 2007.

### Results of occupational health examination

After comprehensive occupation health examination for all workers in the cohort, none was diagnosed as occupational manganism, but some workers complained of insomnia, dizziness, cough, joint pain, and decreased vision with high reported rate. Especially, both rates of insomnia and dizziness were 11.4%, and the symptoms of digestive system including constipation (4.0%), abdominal pain (2.9%), abdominal distension (2.2%), nausea (1.4%) and anorexia (0.4%) were described by workers, but those symptoms like liver area pain, alopecia, increased excitability and asthma, were seldom reported and their reported rates were no more than 1%.

As shown in Table 2, the clinical signs composed of ENT (ears, noses, and throat), internal medicine, surgery department, and nervous system. A total of 284 workers (25.5%) had the abnormal signs of oral cavity including constant anodontia, deep caries, and periodontal disease; 207 workers (18.6%) had the enlargement of superficial lymph nodes, and most of them (96%) occurred in the submandibular lymph nodes. Moreover, 46 subjects (4.1%) were diagnosed as the decrease of muscle strength, whch was the main abnormal sign in the nervous system, but only 1 subject (0.1%) had the increased tremor or abnormal feeling of peripheral nerve. The rate of visual abnormality was 4.3% (48 workers) and other signs were rare with the detection rate of less than 1%.

**Table 2:** The detection rates of anomaly clinical signs in the MEWHC exposed to Mn dust during in the last two weeks

**Signs**	**Number**	**Percent (%)**
**ENT**		
Vision	48	4.3
Hearing	23	2.1
Nasal	4	0.4
Oral cavity^a^	284	25.5
Throat	6	0.5
**Surgery dept**		
Superficial lymph node^b^	207	18.6
Thyroid gland	8	0.7
Skin and mucosa	4	0.4
**Nervous system**		
Muscle strength	46	4.1
Abnormal feeling of peripheral nerve	1	0.1
Increased tremor	1	0.1

^a^ Oral cavity signs included constant anodontia, deep caries, and periodontal abnormality.

^b^ Lymph node enlargement mainly occurred in the submandibular lymph nodes (96%), and the other occured in cervical lymph nodes and supra-clavicular lymph nodes.

### Higher liver enzymes caused by Mn exposure

After adjusting for sex, length of work and smoking habits, our data showed that there were not significant difference for the concentrations of TBILI and IBILI among three exposed groups (*P* = 0.554 and *P* = 0.739, respectively). However, the concentrations of DBILI, ALT and AST were significantly different between these three exposed groups (all *P* < 0.05), respectively. Compared to workers in the low-exposure group, those with the high Mn exposure have higher concentrations of liver enzymes, DBILI: (3.84 ± 1.20) μmol/L vs (3.54 ± 0.85) μmol/L, *P* = 0.006; ALT: (27.04 ± 19.12) IU/L vs (20.38 ± 10.97) IU/L, *P* < 0.001, and AST: (29.96 ± 16.68) IU/L vs (26.39 ± 8.07) IU/L, *P* = 0.007 (Table 3 shown).

**Table 3:** **The difference of liver function between three Mn exposed groups (mean ± SD**)

**Variable**	**Exposure group**			***P***^***a***^	***P***_**trend**_
	**Low (n=381)**	**Intermediate (n=181)**	**High (n=550)**		
TBILI (μmol/L)	11.23±2.20	11.58±3.21	11.48±3.17	0.554	0.231
DBILI (μmol/L)	3.54±0.85	3.83±1.09	3.84±1.20	0.006	0.000
IBILI (μmol/L)	7.64±1.98	7.79±2.45	7.65±2.39	0.739	0.960
ALT (IU/L)	20.38±10.97	21.31±9.75	27.04±19.12	0.000	0.000
AST (IU/L)	26.39±8.07	26.43±6.61	29.96±16.68	0.007	0.000

^a^ Analysis of covariance for differences between the different exposure groups, after adjusting gender, working years and drinking habits.

We used Pearson correlation analysis to explore the trend between exposure group and these damage indices. Table 3 also showed that the concentrations of DBILI, ALT and AST significantly increased with the rise of Mn exposure, and the correlation coefficient was 0.123, 0.182 and 0.125, respectively, all *P* < 0.001. The trend for the other two indices was not found, *P* > 0.05.

### Interaction of drinking and Mn exposure

In order to analyze the interaction of drinking and Mn exposure, all workers were stratified as drinkers and non-drinkers (Table 4). The concentrations of DBILI, ALT and AST were still significantly different between three exposed groups after stratification, and the rising trend was also observed (*P* < 0.05). Meanwhile, there were not significant difference for the concentrations of TBILI and IBILI (*P* > 0.05). Furthermore, those subjects with high Mn exposure and alcohol drinking had the highest levels of liver function indices, especially, shown by the ALT (27.06 ± 19.55) and AST (30.05±17.68), and the significant interaction between drinking and Mn exposure was shown for the DBILI, ALT and AST, respectively, all *P* < 0.01.

**Table 4:** The interaction analysis of Mn exposure and alcohol consumption on workers liver function (mean ± SD)

**Variable**	**Alcohol drinking status**	**Exposure group**			***P***^***a***^	***P***_**interaction**_
		**Low (n=381)**	**Intermediate (n=181)**	**High (n=550)**		
TBILI (μmol/L)	Drinker	11.13±2.12	11.77±3.43	11.54±3.19	0.147	0.376
	Non-drinker	11.34±2.26	11.23±2.76	11.14±3.00	0.828	
DBILI (μmol/L)	Drinker	3.57±0.88	3.89±1.20	3.85±1.18	0.010	0.001
	Non-drinker	3.51±0.83	3.71±0.83	3.84±1.29	0.023	
IBILI (μmol/L)	Drinker	7.52±2.01	7.87±2.63	7.72±2.42	0.425	0.487
	Non-drinker	7.75±1.96	7.64±2.07	7.29±2.20	0.233	
ALT (IU/L)	Drinker	21.05±10.78	21.02±9.70	27.06±19.55	0.000	<0.000
	Non-drinker	19.71±11.14	21.83±9.89	26.85±16.46	0.000	
AST (IU/L)	Drinker	26.77±7.32	26.63±7.17	30.05±17.68	0.008	0.001
	Non-drinker	26.02±8.76	26.16±5.52	29.10±9.54	0.019	

^a^ Analysis of covariance for differences between the different exposure groups in drinkers and non-drinkers, after adjusting for gender and working years.

## Discussion

Chronic low-level exposure to Mn is currently hypothesized as a possible risk factor for the onset of Parkinson's disease [12]. Occupational manganism was closely related to the air Mn concentration in working environment, genetic factors and lifestyles. Crossgrove and Zheng’s study showed that the onset of symptoms of typical manganism appeared from 2 to 34 years’ exposure, average 16.3 years [13]. In this present survey, the average service length among the MEWHC workers was about 13 years. Some technical innovations were gradually carried out to control the Mn level since 2006, and the concentration of Mn has been significantly decreased during the past years, which resulted in no manganism patient or worker with obvious manganism symptoms.

Mn had a special affinity on mitochondrial richly tissues like liver. More than ninety-five percent of intaked Mn was eliminated by billiard excretion [14], which potentially resulted in the direct liver toxicity. Overload Mn in vivo was also probably derived from the obstructive condition affecting the biliary excretion. In fact, multiple mechanisms had been recently proposed to explain hepatotoxic and neurotoxic effects of excess accumulation of heavy metals due to impaired transport or failure of hepatic detoxification [15]. The subclinical liver impairment marked by increase of AST and ALT even likely aggravated the Mn neurotoxicity. For example, rendered Mn exposed individuals at higher risk for Parkinsonism [16]. Meanwhile, the Jordenko’s early survey showed that the serum ALT and AST levels in Mn alloy foundry workers had significant difference compared to the control group [17]. Another recent report described that workers under Mn exposure condition had higher ALT levels and complained more self-conscious symptoms [18]. Our data elucidated the obvious dose–response relationship of liver function abnormality with Mn exposure levels (as shown in Table 3). Among the non-drinkers, the dose-dependency between them was more significant (Table 4). These results supported the hypothesis that Mn exposure caused the direct liver toxicity *in vivo* although further supporting evidence was needed at an individual level.

Alcohol can also lead to adverse effects on liver function, and 90% of uptaken alcohol can be metabolized and decomposed in the liver. Some studies showed that Mn in blood may interact with alcohol use disorders, accentuating neuropsychiatric symptoms [19,20]. Another study suggested that a higher Mn concentration in workers was related with alcoholism [17]. In this present study, 69.5% workers had alcohol drinking habit, which was probably caused by heavy physical labor and over stress. The data also showed that the workers with drinking habit had higher concentrations of liver enzymes than those non-drinkers. The serum indices results indicated that there was a significant interaction between Mn exposure and alcohol consumption. These results suggested that it should be essential to perform the related public health education and inform people to reduce the alcohol drinking amount, especially, for those heavy drinkers.

Some study limitations need be addressed when interpreting these results. First, our investigation only included the drinking habit but not the dose of alcohol consumption. Second, one important confounding factor was the sex ratio between different exposure groups and affected the values of liver function [21], and should be addressed because more women worked in the low exposure group, even though we used the analysis of covariance to adjust the gender variable. Third, the individual cumulative exposure index (CEI) was very important for the better assessment of the dose–response with liver toxicity. We also tried to calculate the index but unfortunately we found the results were imprecise because our obtained monitoring data of air concentration of Mn in various workplaces were not enough, and not all of the working places were included in monitoring spots.

## Conclusions

In this study, we found that both occupational Mn exposure and alcohol consumption can lead to hepatic toxicity, especially, and they may interact with each other, accentuating the damage representing by three serum indices of liver function. It is necessary to control drinking habit for the occupational Mn exposed workers and exam liver function in the prospective occupational health examination.

## Abbreviations

MEWHC: Manganese exposure workers healthy cohort; Mn: Manganese; TBILI: Total bilirubin; DBILI: Direct bilirubin; IBILI: Indirect bilirubin; ALT: Alanine transaminase; AST: Aspartate transaminase; ENT: Eyes, nose and throat.

## Competing interests

All authors declare that they have no competing interests.

## Authors’ contributions

YXB and ZYF conceived and designed the study and cohort. LJ, LQ, CKC, LZF, SYF, and YPY performed the investigation. DQ and LZF analyzed the data. DQ and YXB Writed the paper. All authors read and approved the final manuscript.
